# IgE-Binding and Immunostimulating Properties of Enzymatic Crosslinked Milk Proteins as Influenced by Food Matrix and Digestibility

**DOI:** 10.3390/nu14214584

**Published:** 2022-11-01

**Authors:** Sara Benedé, Mónica Martínez-Blanco, Rosina López-Fandiño, Elena Molina

**Affiliations:** Instituto de Investigación en Ciencias de la Alimentación (CIAL, CSIC-UAM), Nicolás Cabrera 9, 28049 Madrid, Spain

**Keywords:** milk allergens, casein, whey protein, transglutaminase, allergenicity, digestion

## Abstract

Dairy foods are essential in the diet, although in some susceptible individuals they may cause allergy to cow’s milk proteins. Therefore, alternative methods are sought to reduce their allergenicity. Transglutaminase (TG) is widely used in dairy products mainly to improve texture. Although it has been claimed that TG can be used to modify the digestibility and allergenicity of foods, its impact within a real matrix has been rarely studied. The aim of this work was to assess the allergenic potential of crosslinked skim milk (SM), milk casein fraction (CN), and whey protein (WP). To this purpose, inhibition ELISA with sera from milk allergic patients, in vitro activation tests of mouse mast cells and splenocytes, and simulated gastrointestinal digestion assays were performed. The results showed that cross-linking increased the binding of IgE to WP, but decreased IgE-binding to SM and CN. However, no differences were observed in the ability of cross-linked proteins to induce mast cell degranulation compared to native proteins. The cross-linking of SM and CN reduced Th2 cytokine release from the splenocytes of sensitized mice. All TG-treated samples exhibited more resistance to in vitro digestion than the untreated proteins and the human IgE binding capacity after digestion was higher. In conclusion, TG treatment of milk proteins does not reduce the risk of eliciting allergic symptoms in cow’s milk allergic patients.

## 1. Introduction

Food allergies are increasing around the world with unprecedented complexity and severity. They constitute a serious health problem that causes large public health costs and severely deteriorates the quality of life of patients. Of all known food allergies, cow’s milk allergy is of special interest due to its high incidence during the first three years of life [1]. Mainstay milk allergy treatment consists of avoiding all foods containing milk proteins, nutritional counseling, as many foods cannot be consumed, and emergency treatment to reduce the allergic response. However, avoidance of milk proteins involves quite a few dietary restrictions since they are used as ingredients in many products beyond dairy foods. Furthermore, the positive contribution of milk proteins and related bioactive peptides to a healthy diet has been increasingly documented [2] and the lack of some micronutrients present in dairy foods, such as calcium, can also have other (deleterious) effects on the growth and development of children [3].

Different approaches have been used to try to produce hypoallergenic foods, taking advantage of the transformations that food components undergo during processing [4]. Among them, attention has recently been paid to protein cross-linking with transglutaminase, an enzyme that catalyzes the formation of isopeptide bonds between the amino acids glutamine and lysine in proteins, in part because it has been accepted in the European Union for the marketing of food products. Its most widespread use includes the manufacture of cheese and other dairy products, meat processing, production of edible films, and manufacture of bakery products. Studies on the crosslinking of food proteins with TG mainly evaluate its influence on physical properties of food, such as solubility, gelling, or emulsifying capacity, among others. Its contribution to the reduction of allergenicity has also been assessed [5]. However, beyond some promising results, the optimal conditions for its use have not yet been defined, nor is there consensus on its application by the food industry.

Partial hydrolysis of whey proteins or casein followed by TG-catalyzed crosslinking of the hydrolysates was proposed as a strategy to produce protein polymers with lower antibody binding properties but with improved textural and sensory properties [6,7]. Regarding the intact proteins, TG-induced polymerization was shown to decrease the antigenicity of whey protein and casein preparations [7] and TG-polymerized β-Lg gave negative prick tests in allegic subjects [8]. However, the assessment of the sensitizing and eliciting capacity of polymerized milk proteins in vivo revealed that TG-treated caseinate exhibited a somehow lower sensitizing potential but similar eliciting potential relative to its non-modified counterpart, while cross-linked β-Lg elicited a stronger allergic sensitization and a lower allergic effector response [9,10]. This different behavior was attributed to the enhanced resistance to proteolysis of cross-linked β-Lg, which redirected protein transport to PP and changed uptake and processing by antigen presenting cells [10]. Therefore, the relevance of protein cross-linking in the case of food allergy needs to be studied by taking into account the real food matrix that contains components with different resistance to processing and gastroduodenal digestion, factors which ultimately allow them to interact with the intestinal mucosa where absorption occurs [4].

The aim of this work was to assess the effect of TG-mediated crosslinking on the IgE-binding, ability to induce mast cell degranulation, and immunostimulating properties of milk in comparison with its two main protein fractions. Moreover, a physiologically relevant in vitro digestion model was used to investigate the susceptibility to digestion of the cross-linked milk proteins and the binding of human IgE to the resulting digests.

## 2. Materials and Methods

### 2.1. Crosslinking of Milk Proteins

Milk, obtained from the local farm La Colmenareña (Madrid, Spain), was skimmed by centrifugation, 3000× *g* at 4 °C for 30 min, filtered through glass wool, lyophilized, and stored at −20 °C. Commercial micellar casein Prodiet 85B (CN) was from Ingredia S.A, (Arras, France). Whey protein isolate (WP) (LACPRODAN DI-9224K) was from Arla Foods Ingredients (Sønderhøj, Denmark). Protein concentration of skim milk (SM), CN, and WP was determined by the Kjeldahl method [11]. The absence of lipopolysaccharide (LPS) was confirmed using the transfected cell line THP1-XBlue™ and the QUANTI-Blue™ assay (Invitrogen, Carlsbad, CA, USA), following the manufacturer’s instructions. Protein crosslinking was performed with TG (MTG-Activa ^®^-MP, Ajinomoto Co., Sao Paulo, Brazil; enzymatic activity:100 U/g), a commercial powder including 1% TG and 99% maltodextrin. The enzyme was dissolved in PBS and mixed (1:1, *v/v*) with the SM, CN, and WP solutions to achieve a final protein concentration of 2.5 mg/mL and a final enzyme activity of 1, 10, 100 and 1000 U in the reaction mixture. TG cross-linking experiments were carried out at 40 °C for 1, 3, 8, and 24 h. Then, samples were heated at 90 °C for 10 min for enzyme inactivation.

### 2.2. Electrophoresis SDS-PAGE

Electrophoresis was performed in a Criterion^TM^ cuvette on preform bis–tris gels (12%) at 120 V, using XT-MES as running buffer. Samples were diluted in sample buffer with 5% (*v/v*) β-mercaptoethanol to a final protein concentration of 250 µg/mL, then heated at 95 °C for 10 min. Hence, 30 μL of sample were placed into each lane. Staining was completed with Bio-Safe Coomassie G-250. All equipment and reagents were from Bio-Rad (Richmond, CA, USA).

### 2.3. Human IgE Binding by Inhibition ELISA

Human IgE-binding to native and TG cross-linked SM, CN, and WP and their digests was evaluated by inhibition ELISA [12], using a pool of 7 different sera from milk allergic patients with levels of IgE specific to α-LA, β-LG, and CN described in Table 1. To perform ELISA, the final protein concentration was 2.5 mg/mL and the final enzyme activity in the mixture was 100U, maintaining the reaction for 24 h.

### 2.4. Bone Marrow Derived Mast Cells

Bone marrow-derived mast cells (BM-MCs) were in vitro differentiated into a mucosal-like phenotype as described previously [13]. Briefly, bone marrow cells were collected from femurs and cultured in DMEM with glucose and L-glutamine, supplemented with 10% fetal bovine serum, penicillin/streptomycin, and sodium pyruvate (Biowest, Nuaillé, France), plus stem cell factor, IL-3, IL-9 and TGF-β (Peprotech, Rocky Hill, NJ, USA), for a minimum of 4 weeks and a maximum of 8 weeks before being used. For activation through cross-linking of the IgE receptor, MCs (1 × 10^6^ cells) were sensitized with serum from BALB/c mice orally sensitized with 5 mg of SM, CN, and WP plus 10 μg of cholera toxin (List Biologicals, Campbell, CA, USA) [14]. After washing, MCs were activated with samples in HEPES degranulation buffer. To study the activation through an IgE independent pathway, non-sensitized MCs were also stimulated with samples. β-hexosaminidase was detected by an enzymatic colorimetric assay [15]. The percentage of β-hexosaminidase release was calculated considering the total β-hexosaminidase content.

### 2.5. Cell Viability of Mice Splenocytes

The cell viability assay was carried under the culture conditions described below and determined by 3-(4,5-dimethylthiazol-2-yl)-2,5-diphenyltetrazolium bromide (MTT) method. Hence, 20 μL of MTT solution (5 mg/mL PBS) were added to each well. Samples were incubated for a further 4 h in dark. Then, media were removed, and 100 μL of dimethyl sulfoxide:methanol (1:1) was added to dissolve the purple insoluble MTT formazan, and the absorbance was measured at 570 nm in a Fluorostar Optima microplate-reader (BMG LabTech, Ortenberg, Alemania) using a 650 nm filter as a reference.

### 2.6. Cytokine Secretion of Mice Splenocytes

Splenocytes were obtained from female BALB/c mice orally sensitized with SM, CN, and WP (*n* = 5). After sacrifice, whole spleens were excised, and cell suspensions were prepared. Splenocytes were cultured in RPMI 1640 plus 10% of fetal bovine serum, L-glutamine (2 mM), penicillin (50 U/mL) and streptomycin (50 μg/mL) (all from Biowest) at 4 × 10^6^ cells/mL in 48-well plates. Stimuli consisted of 200 μg/mL of untreated or TG cross-linked SM, CN, WP, and PBS as negative control. Control and stimulated splenocyte cultures were maintained for 72 h at 37 °C in a 5% CO_2_ gassing incubator and were performed in duplicate. The supernatants were collected and kept frozen (−80 °C) until assayed for IL-4 and IL-13 quantification using mouse ELISA kits (eBioscience, San Diego, CA, USA).

### 2.7. In Vitro Gastrointestinal Digestion

Simulated gastrointestinal digestion of native and TG cross-linked SM, CN, and WP was performed as described previously [16]. Briefly, a gastric phase was conducted with porcine pepsin (EC 3.4.23.1, 4220 U/mg, Sigma Aldrich, St. Louis, MO, USA), at a final concentration of 182 U/mg of protein for 1 h at 37 °C adjusted to pH 2.0 (2 M HCl). The reaction was stopped by raising the pH to 7.0 (2 M NaHCO3). The duodenal phase was carried out using gastric digests by adding Corolase PP (AB Enzymes, Darmstadt, Germany) at an enzyme:substrate ratio of 1:25 (*w/w*), and incubating for 30 min at 37 °C. Stopping of the reaction was achieved by heating at 95 °C for 10 min.

### 2.8. RP-HPLC

RP-HPLC analysis was conducted on a Waters 600 HPLC instrument (Waters, Mildford, MA, USA) with a RP318 column (250 × 4.6 mm, 5 µm of particle size, 300Å pore size, Bio-Rad). 100 µL (2 mg/mL) of sample were injected and eluted for 60 min using 0.37% (*v/v*) trifluoroacetic acid from Scharlau Chemie (Barcelona, Spain) in double-distilled water as phase A, and 0.27% (*v/v*) trifluoroacetic acid in HPLC-grade acetonitrile (Lab-Scan, Gliwice, Poland) as phase B. The gradient was linear, increasing B in A from 0% to 60% at a flow rate of 1 mL/min during 90 min, followed by a 35-min wash with 60% and 10 min with 100% of solvent B. Detection was at 214 nm and data were processed by using Empower 2 Software from Waters.

### 2.9. Statistical Analysis

Statistical analyses were performed using GraphPad Prism software, version 7.0e (GraphPad). The two-tailed Student’s *t* test was used for determining statistical significance (*p* < 0.05). Data are expressed as the mean ± SEM of 3 independent experiments.

## 3. Results and Discussion

### 3.1. Protein Polymerization

**Figure 1:**
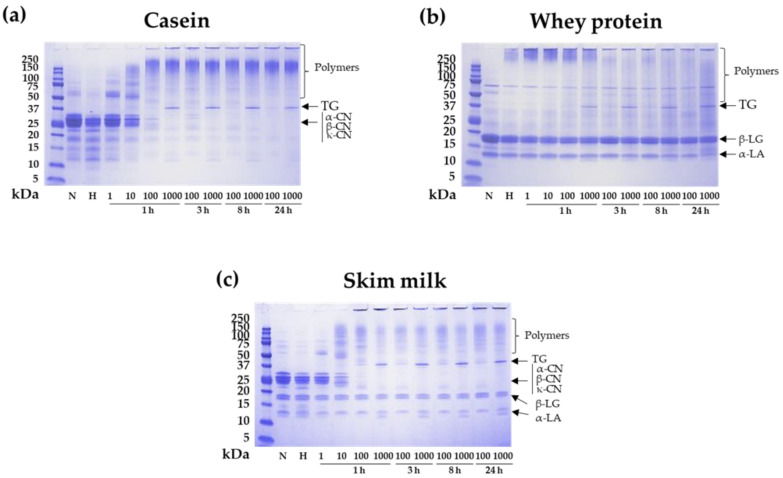
Electrophoretic patterns of TG treated samples. SDS-PAGE using Bis-Tris 12% acrylamide gels of (**a**) casein, (**b**) whey proteins and (**c**) skim milk after cross-linking using transglutaminase (TG) at 1, 10, 100, and 100 U of TG/g of protein during 1, 3, 8, and 24 h of incubation at 40 °C. Heating to 90 °C for 10 min to deactivate the TG was applied to the samples. Native (N) and heated (H, 90 °C for 10 min) proteins were included as control samples. α-casein = α-CN, β-casein = β-CN, k-casein = k-CN, β-lactoglobulin = β-LG and α-lactalbumin = α-LA.

CN, WP, and SM were susceptible to TG mediated cross-linking (Figure 1). As expected, increasing TG doses and incubation times led to higher polymerization degrees. The casein fraction was the most susceptible to polymer formation due to its little secondary and tertiary structure, flexible and random-coil arrangement, and absence of any disulphide bonds in α_S1_- and β-CNs, which allow a greater exposure of reactive groups and an increased accessibility to the enzyme [17]. The presence of protein aggregates corresponding to dimers and trimers was observed after 1 h of CN polymerization with 1 and 10 U of TG/g. After longer incubation periods, higher polymers were detected, and no presence of monomers was observed at 24 h (Figure 1a). The polymerization of β- and κ-CNs occurs earlier than that of the other milk proteins, as also observed by other authors [18]. Monomers of β-LG and α-LA were still detected after 24 h of treatment with TG, even at the highest concentration used (1000 U/g of protein) (Figure 1b). WP, due to their compact globular structures, cross-linked less efficiently [17]. Although there is no specific legislation about minimum or maximum quantities of TG allowed to be added to food products in the European Union or in the United States, most studies that addressed the use of TG in dairy foods use a maximum of 100 U/g of protein [19,20]. We evaluated the use of 1000 U/g of protein to assess whether higher enzyme activity could result in higher crosslinking efficiency, but although polymerization increased slightly compared to 100 U/g, it was not enough to convert all monomeric proteins into polymers. The dairy matrix did not change the susceptibility to cross-linking of the main milk fractions, as similar effects were observed in milk and in the isolated proteins (Figure 1c).

### 3.2. Influence of Crosslinking on IgE-Binding Capacity

**Figure 2:**
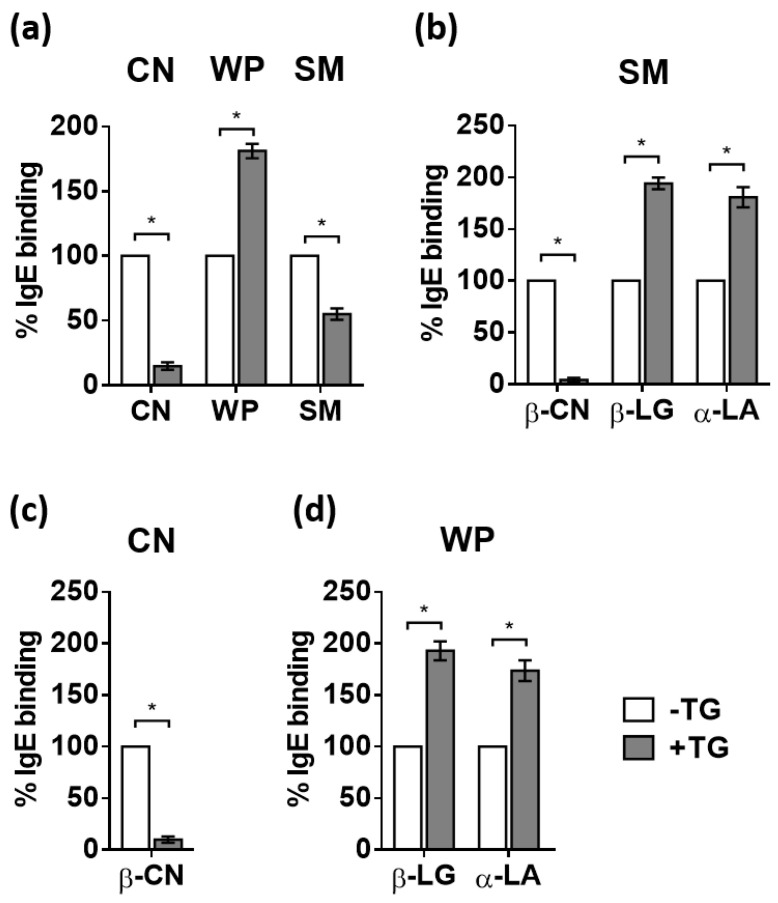
IgE binding to casein (CN), whey proteins (WP) and skim milk (SM), either untreated (−TG, white bars) or treated with transglutaminase (+ TG, grey bars), estimated by inhibition ELISA with sera from milk allergic patients (Table 1). (**a**) IgE binding to CN, WP, and SM, (**b**) specific IgE binding to β-casein (β-CN), β-lactoglobulin (β-LG), and α-lactalbumin (α-LA) in SM; (**c**) specific IgE binding to β-CN in CN, and (**d**) specific IgE binding to β-LG and α-LA in WP. The proteins used for pre-coating the plates are indicated on the x-axes. Data are expressed as means ± SEM of 3 different experiments. Statistically significant differences (*p* < 0.05) are indicated by asterisks.

IgE reactivity of pooled sera from milk allergic patients from our serum library (Table 1) towards intact and polymerized samples was assessed by inhibition ELISA. Cross-linking of CN and SM led to a reduction in their IgE binding capacity. However, after treatment with TG, IgE binding to WP increased (Figure 2a), probably due to the exposure of hidden epitopes due to conformational changes in the protein [21]. Other allergenic proteins or extracts, such as ovalbumin [22], Ara h 1 [23], tropomyosin [24], β-LG [20], and wheat flour [25], have shown a reduced allergenicity after cross-linking with TG. However, peanut [26] and wheat [27] proteins showed higher IgE binding capacity after cross-linking with TG. These results highlight the importance of the substrate type in the IgE binding capacity of the TG reaction products. 

The contribution of individual milk proteins to the binding of IgE capacity to SM, CN, and WP was assessed by inhibition ELISA pre-coating the plates with either β-CN, β-Lg, or α-la. In SM, the binding of IgE to β-CN was greatly reduced after treatment with TG to 3% of the IgE binding to β-CN in the untreated sample (Figure 2b). However, the specific IgE binding to β-LG and α-LA increased to 194% and 181%, respectively. This is consistent with the data obtained when specific inhibition ELISA was performed on CN and WP samples (Figure 2c,d). Different results were found by Fotschki et al., who showed that polymerization with TG decreased β-LG immunoreactivity and increased IgE binding of α-CN [20]. However, they used polyclonal rabbit antibodies, obtained from rabbits immunized against commercial purified allergens instead of human sera from naturally sensitized allergic patients. Villas-Boas et al. [19] also showed that TG crosslinking decreased the potential antigenicity of β-LG by modifying or hiding epitopes, although cross-linking was performed in the presence of cysteine.

**Figure 3:**
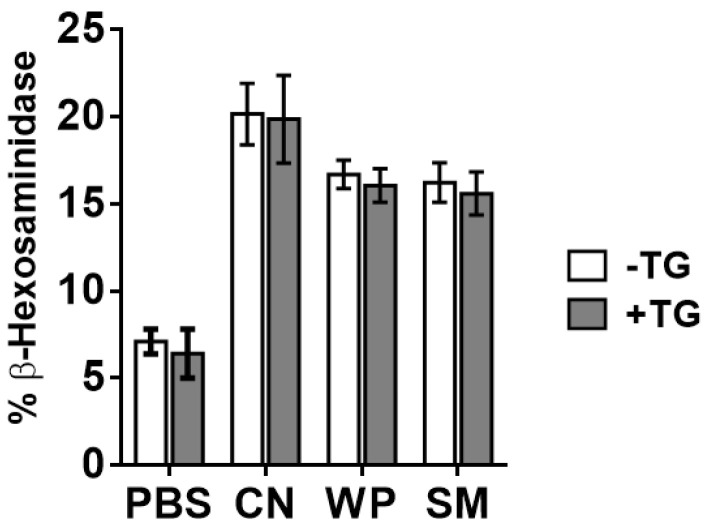
Mast cell degranulation capacity. Measurement of β-hexosaminidase release induced by casein (CN), whey proteins (WP) and skim milk (SM), either untreated (white bars) or treated with TG (grey bars), after sensitization of bone marrow derived mast cells with sera from CN, WP and SM allergic mice, respectively. Data are expressed as mean ± SEM of 3 different experiments.

Because mast cells are the main effector cells of the allergic response, we assessed the ability of TG-treated SM, CN, and WP to activate them through an IgE dependent pathway using BM-MCs (Figure 3). Cells were primed with sera from mice sensitized to SM, CN or WP and then treated with specific antigens or PBS. No differences were observed in the percentage of β-hexosaminidase released by BM-MC after incubation with TG-treated SM, CN, and WP compared to cells stimulated with the untreated proteins. To rule out that cell activation occurred through a non-IgE-mediated pathway, we confirmed that neither the protein samples treated with TG nor TG alone induced degranulation in non-sensitized cells. These data would indicate that, although the IgE-binding capacity of milk proteins is affected by the action of TG, the effector cellular response may not be altered. A similar behavior was observed by Nakamura et al. [28], who observed a pronounced increase in the IgE reactivity of a hydrolyzed wheat protein treated with TG without affecting the IgE response of patients with wheat allergy.

### 3.3. Immunostimulating Properties of Crosslinked Milk Proteins

**Figure 4:**
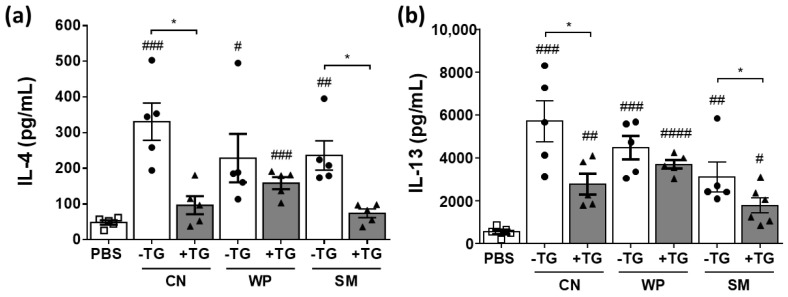
Stimulation of splenocytes. Th2 cytokines (**a**) IL-4 and (**b**) IL-13 released from cultures of spleen cells from casein (CN), whey protein (WP), and skim milk (SM) sensitized BALB/c mice (*n* = 5) stimulated with CN, WP, and SM, either untreated (white bars) or treated with TG (grey bars). Pounds and asterisks indicate, respectively, statistically significant differences with respect to cells stimulated with PBS or between both experimental groups. Data are expressed as mean ± SEM. # and * *p* < 0.05, ## *p* < 0.01, ### *p* < 0.001, #### *p* < 0.0001.

The immunostimulating properties of TG-treated SM, CN, and WP were evaluated in splenocytes from SM, CN, and WP sensitized BALB/c mice, respectively (Figure 4). Stimulation of spleen cells with untreated CN, WP, and SM led to an increased Th2 cytokine production compared with control cells (PBS). Cytokine secretion from cells stimulated with TG and PBS was similar (not shown), indicating an antigen specific stimulation. Cross-linking of SM and CN reduced the release of IL-4 from the splenocytes to a level similar to that of control cells. IL-13 secretion was also reduced, although it was not significantly different with respect to the control cells. In contrast, IL-4 and IL-13 production in splenocytes stimulated with WP was similar regardless of whether WP was cross-linked or not. Th2 cytokines (IL-4, IL-5, and IL-13) secreted by differentiated and expanded T-helper lymphocytes favor the activation of B lymphocytes that differentiate into plasma cells, producing large amounts of IgE antibodies, whose activation on the surface of basophils and mast cells triggers the allergic response [29]. The lower levels of IL-4 and IL-13 detected in the supernatants of splenocytes incubated with TG-treated SM and CN, but not WP, may indicate that crosslinked SM and CN exert positive immunosuppressive effects.

Some studies have determined the influence of crosslinking on the allergenic properties of proteins through the analysis of the IgE binding capacity by ELISA, Western blotting, or degranulation tests using basophil and mast cell cultures [30,31,32], but very few have determined their impact on other immune cells. Bai et al. [33] observed that TG cross-linked tofu proteins reduced the levels of Th2 cytokines released by spleen cells from mice allergic to tofu protein cultured in vitro compared to native proteins. Similarly, ovalbumin and egg white subjected to high-pressure treatment and crosslinking (simultaneously or thereafter) induced a lower IL-5 and IL-13 release from splenocytes [22].

**Figure 5:**
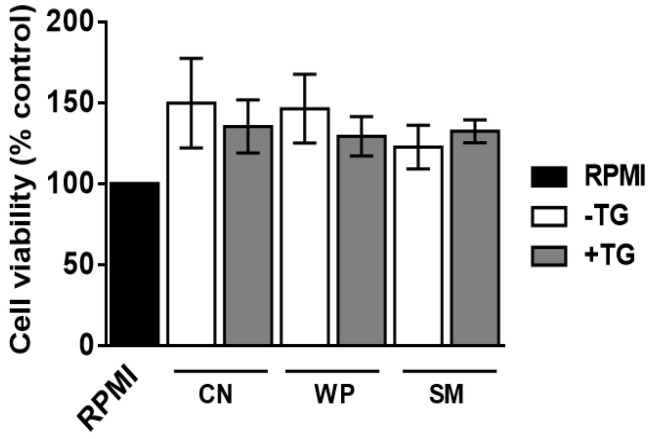
Cytotoxicity assay. The MTT method was used to determine the viability of spleen cells from casein (CN), whey protein (WP), and skim milk (SM) sensitized BALB/c mice cultured with either untreated (white bars) or TG cross-linked CN, WP, and SM (grey bars).

None of the samples analyzed in this study exhibited spleen cell cytotoxicity after a 72 h-culture (Figure 5), indicating that the inhibitory effects on cytokine release caused by TG were due to a lower reactivity of the cross-linked proteins. Other in vivo and in vitro studies of toxicity of microbial TG have revealed safety concerns about its food applications [34] and therefore more research is need in this regard.

### 3.4. Impact of Crosslinking on Digestibility of Milk Proteins

**Figure 6:**
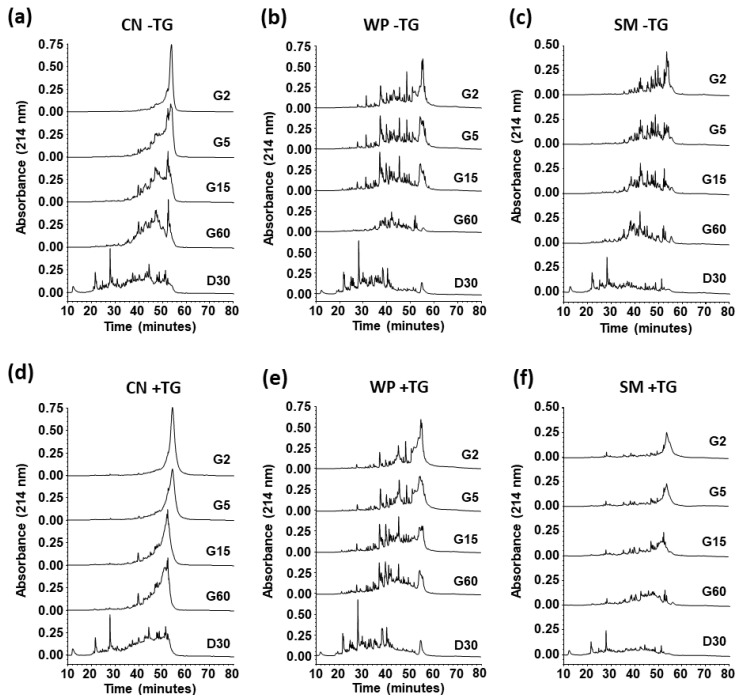
Digestibility of TG cross-linked milk proteins. RP-HPLC patterns of untreated (−TG; **a**–**c**) and TG cross-linked (+TG; **d**–**f**) casein (CN; **a**,**d**), whey proteins (WP; **b**,**e**), and skim milk (SM; **c**,**f**), after 2 (G2), 5 (G5), 15 (G15), and 60 (G60) min of simulated gastric digestion with pepsin, and subsequent duodenal digestion with Corolase PP for 30 min (D30).

The resistance to in vitro gastric digestion of TG treated SM, CM, and WP was higher than that of the native proteins, and this was especially noticeable during the first minutes of pepsin treatment (Figure 6). Resistance to digestion could be used as a predictive factor in assessing the allergenic potential of a protein [35] and, in this respect, it is essential to evaluate the digestion process of proteins within the food matrix in which they are usually found [20]. As shown in Figure 6, digestion with pepsin did not lead to complete hydrolysis, as there was intact protein in all cases, while SM and CN were almost completely hydrolyzed with Corolase PP. In the case of WP, intact protein was still observed after 30 min of simulated duodenal digestion in both native and TG-treated proteins, although the peak corresponding to intact proteins was higher in the cross-linked sample.

The peptide profile generated during digestion in the gastric and duodenal phases was similar in the native samples and in those treated with TG. Similar results were found by Fotschki et al., who observed that TG influenced the gastric digestibility of cow and horse milk, slowing digestion of the proteins [20]. Cross-linked β-LG was found to be more resistant to simulated gastrointestinal digestion [10]. In contrast, polymerization of chemically denatured β-LG with TG was reported to facilitate the action of gastrointestinal enzymes [32]. Similarly, Havenaar et al. [36] indicated that caseinate treated with TG was more digestible than the untreated protein. These discrepancies may be due to the use of different polymerization conditions and in vitro digestion protocols, as it is well known that the rate of digestion of polymerized proteins depends on the type of structure generated [37].

As expected, in vitro gastrointestinal digestion significantly reduced the IgE-binding capacity of SM, CN, and WP (Table 2). The concentration (μg/mL) for 50% of the maximal IgE binding, also known as EC50, of SM and CN treated with TG after gastric and duodenal digestion was approximately an order of magnitude higher than that of the digests for the native samples and similar results were obtained for WP samples. These data are in agreement with the lower digestibility of samples cross-linked with TG observed by RP-HPLC (Figure 6).

## 4. Conclusions

TG was effective in crosslinking SM, CN, and WP, forming high molecular weight polymers, although a substantial amount of unmodified monomeric serum proteins remained. Although the binding of human IgE to β-CN was significantly reduced and that to β-LG and α-LA was increased after treatment of SM, CN, and WP with TG, no differences were observed in either case in the ability to induce mast cell degranulation compared to the untreated proteins. SM and CN subjected to cross-linking induced a lower release of Th2 cytokines from splenocytes of mice sensitized, respectively, to SM and CN, but WP treated with TG did not modify the production of IL-4 and IL-13 with respect to the native protein in spleen cells from WP-sensitized mice. TG-treated SM, CM, and WP were more resistant to digestion than the untreated proteins and, consequently, the binding of human IgE to their digests was also higher. These results question the use of crosslinking with transglutaminase to obtain hypoallergenic products.

## Figures and Tables

**Table 1:** Sera used in the study. Specific IgE levels (kU/L) towards casein (CN), α-lactalbumin (α-LA), β-lactoglobulin (β-LG) and cow’s milk of the human sera used in the inhibition ELISA and mast cell activation assay.

Serum	IgE (kU/L)			
	CN	α-LA	β-LG	Cow’s Milk
1	40.0	12.1	8.3	48.2
2	16.1	5.3	7.8	23.2
3	<100	<100	<100	<100
4	73.2	29.8	5.0	54.1
5	20.5	19.2	7.6	20.1
6	>100	>100	>100	>100
7	62.7	>100	>100	>100

**Table 2:** IgE binding to untreated and TG cross-linked slim milk (SM), casein (CN), and whey proteins (WP), before and after gastric and duodenal digestion, as estimated by inhibition ELISA with sera from milk allergic patients (EC50, µg/mL).

	Undigested		Gastric		Duodenal	
	−TG	+TG	−TG	+TG	−TG	+TG
Casein	0.11	0.79	35.50	4.96	289.23	21.19
	(+/−0.01)	(+/−0.15)	(+/−5.81)	(+/−0.42)	(+/−14.48)	(+/−3.92)
Whey protein	0.74	0.41	1042.59	87.33	1236.17	49.45
	(+/−0.03)	(+/−0.01)	(+/−107.98)	(+/−10.17)	(+/−112.38)	(+/−7.05)
Skim milk	0.39	0.72	99.26	23.81	31,470.27	9188.28
	(+/−0.01)	(+/−0.06)	(+/−12.32)	(+/−3.48)	(+/−3567.51)	(+/−442.36)

Values are the mean of 3 independent experiments (standard deviation in brackets).

## Data Availability

Not applicable.
